# Short-term Outcomes of Saffron Supplementation in Patients with Age-related Macular Degeneration: A Double-blind, Placebo-controlled, Randomized Trial

**Published:** 2016

**Authors:** Alireza LASHAY, Gholamreza SADOUGH, Elham ASHRAFI, Mohammadreza LASHAY, Morteza MOVASSAT, Shahin AKHONDZADEH

**Affiliations:** 1 Farabi Eye Research Center, Tehran University of Medical Sciences, Tehran, Iran; 2 Psychiatric Research Center, Roozbeh Hospital, Tehran University of Medical sciences, Tehran, Iran

**Keywords:** Saffron Supplementation, Age-related Macular Degeneration, Randomized Trial

## Abstract

In modern pharmacological medicine, saffron is used for various purposes due to its antioxidant effect. This study evaluated retinal function after treatment with saffron supplementation during a follow-up period of 6 months to provide further insight into the efficacy and safety considerations of this treatment. Sixty patients with wet or dry age-related macular degeneration (AMD) were randomly assigned to receive oral saffron 30 mg/d or placebo supplementation for 6 months. Optical coherence tomography (OCT), electroretinography (ERG), fluorescein angiography, and visual acuity testing were performed at baseline and 3 and 6 months after treatment. The main outcome measures were OCT, ERG amplitude, and implicit time. Six months after treatment, no statistically significant decrease in OCT results was observed between the groups with dry AMD (P = 0.282). However, there was a statistically significant increase in ERG results between the groups at 3 months after treatment (P = 0.027). In addition, there was a significant decrease in OCT results between groups with wet AMD at the follow-up (P = 0.05). Finally, there was a significant increase in ERG findings between the groups with wet AMD at 3 months after treatment (P = 0.01), but these changes decreased at 6 months after treatment (P = 0.213). Daily supplementation with 30 mg of saffron for 6 months may result in a mid-term, significant improvement in retinal function in patients with AMD.

## Introduction

In western countries, age-related macular degeneration (AMD) is the leading cause of visual impairment in people aged 55 years or older, (1-5) and the prevalence of this disease is expected to increase globally because of demographic and epidemiologic transitions (6-8). None of the current therapeutic strategies optimally improve visual acuity, especially in those with the neovascular form of AMD (8-12); moreover, results are usually unsatisfactory and require frequent retreatments (9-12). Although numerous medical and surgical modalities for AMD are under investigation, none have yet provided satisfactory effects regarding the development of advanced AMD (13-17). The use of herbal drugs by patients has considerably increased in the past few decades, and physicians’ interest in these agents is increasing owing to the fewer side effects, better tolerability, and equal efficacy compared to conventional medications. Before such alternative therapies can be used ethically or effectively, however, it is crucial to examine traditional herbal compounds through the lens of modern scientific investigation. *Crocus sativus L.,* commonly known as saffron, is a perennial stemless herb of the Iridaceae family that principally grows in Mediterranean countries, especially Iran (18). Modern pharmacological studies have demonstrated that saffron extract and its active constituents may have anti-tumor, radical scavenger, memory-enhancing, and hypolipidemic properties (18-22). Recent research has highlighted the importance of antioxidant therapy in preventing oxidative stress-induced neuronal damage, and it has been hypothesized that the saffron extract may be a useful treatment option for AMD because of its powerful antioxidant effects (23-25). In addition to its antioxidant and anti-inflammatory properties, studies have suggested that it may protect against retinal stress (26, 27). Results from the Age-Related Eye Disease Study (AREDS) showed that antioxidant supplementation may prevent or lessen the development of AMD in any stage of the disease (28). Several potential antioxidants are not studied in current clinical trials; therefore, we decided to study the efficacy and safety of saffron supplementation with respect to retinal function and AMD treatment using a placebo-controlled, double-blind, randomized controlled trial.

## MATERIALS AND METHODS

**Figure 1:**
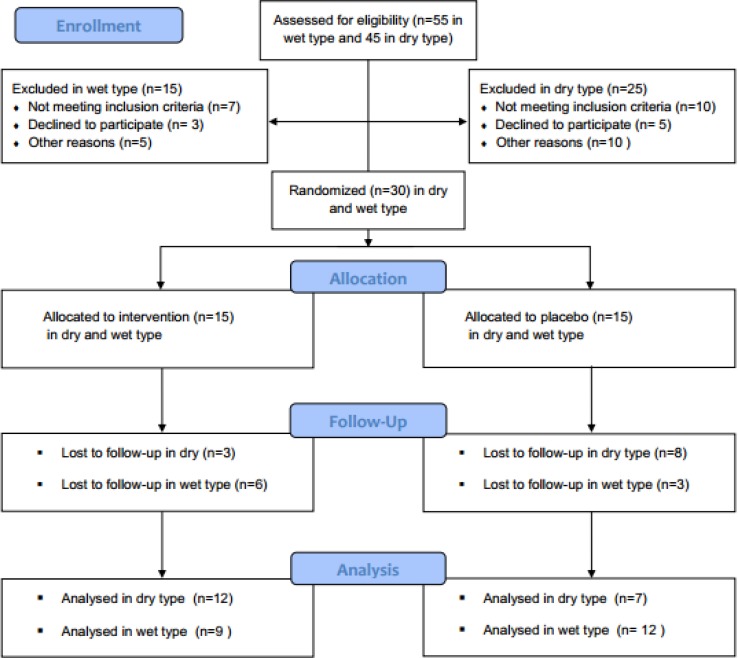
CONSORT 2010 flow diagram

This randomized, double-blind, placebo-controlled study was approved by the Ethics Committee of the Eye Research Center at Farabi Eye Hospital and has been registered in the Iranian National Registry for Clinical Trials (IRCT 201205219820N1). Signed informed consent was obtained from all participants before enrolment. Thirty patients with dry AMD and 30 with wet AMD were recruited from the Retina Clinic at Farabi Eye Hospital between June 2011 and March 2012. Inclusion criteria were a minimum age of 65 years, a physical status class of I-II based on the American Society of Anaesthesiologists classification system, a clinical diagnosis of dry or wet AMD confirmed by fluorescein angiography, best corrected visual acuity between 20/400 and 20/40 in the study eye, and clear optical media. Patients were excluded if they had cataracts, glaucoma, corneal opacities, any sign of retinal or optic nerve disease other than AMD, or systemic disease. A patient flowchart according to the CONSORT statement is summarized in Figure 1.

All participants with wet AMD received monthly intravitreous bevacizumab (IVB) injections for 3 months according to the standard protocol of the American Academy of Ophthalmology (29). Patients were then randomly allocated to either the treatment group (saffron group) or the control group (placebo group) using computer-generated numbers in sealed envelopes. The saffron group received a daily dose of 2 oral saffron capsules (Saffro Mood), each containing 15 mg of saffron extract, donated by Green Plants of Life Co. (IMPIRAN; Tehran, Iran) for 6 months. Control patients received placebo capsules, shaped similarly to the saffron capsules, with the same dose and duration. Patients were not permitted to consume other nutrients or supplements that could affect outcome measures during the study period. Patient compliance was checked weekly by telephone. For all patients, optical coherence tomography (OCT), electroretinography (ERG), and fluorescein angiography (FA) were performed at baseline and at 3- and 6-month follow-up visits. Primary outcome measures were based on macular thickness and ERG amplitude values. During the entire course of the trial, no other systemic pharmacologic agents were administered. All examinations and assessments were performed by a single ophthalmologist. All individuals involved in the study, including those who administrated the medications and performed the examinations, as well as the enrolled patients, were blinded to the assigned treatment groups as each treatment group was referred to according to a binary code.

Statistics

Data were analyzed using SPSS software version 21. Quantitative data were analyzed using the student t-Test and are described as the mean ± SD. The effect of time was assessed using repeated measures analysis of variance (repeated measures ANOVA). A P-value < 0.05 was considered statistically significant.

## RESULTS

**Figure 2:**
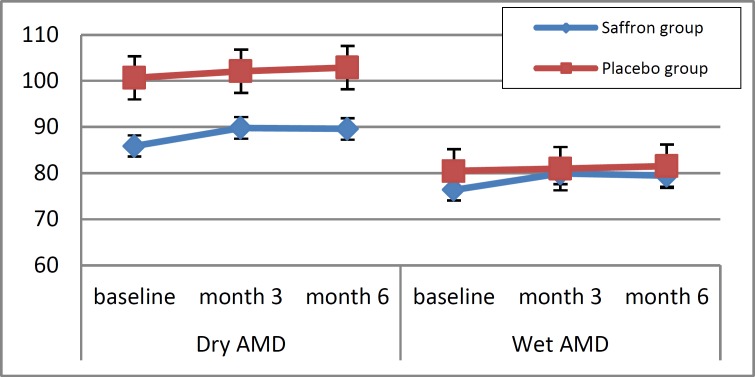
Trend of changes in macular thickness (micron) from baseline to 6 months in the treatment (saffron) and control (placebo) groups of patients with dry and wet age-related macular degeneration (AMD).

Of the 60 participants who initially enrolled, 40 patients completed the study: 21 patients in the saffron group (12 patients with dry AMD and 9 with wet AMD) and 19 patients in the placebo group (7 patients with dry AMD and 12 patients with wet AMD). The enrolled patients included 16 women and 24 men, and there were no significant gender differences between the saffron and placebo groups (P = 0.583). Mean age did not differ significantly between saffron and placebo groups for patients with dry AMD (68.4 ± 4.7 and 63.0 ± 6.8 years, respectively, P = 0.52) or wet AMD (66.8 ± 5.7 and 69.3 ± 9.2 years, respectively, P = 0.56). Additionally, 7 patients were smokers and there was no significant inter-group difference in the number of smoking patients (P = 0.426). Table 1 summarizes our study findings in terms of macular thickness and ERG amplitude in each study group as well as the results of inter-group comparisons using independent t-tests and repeated measures ANOVA. Six months after treatment began, changes in macular thickness were significantly different between the saffron and placebo groups for patients with wet AMD (P = 0.05) but not those with dry AMD (P = 0.28). The trend for changes in macular thickness is illustrated in Figure 2. ERG amplitude changes were significantly different between the saffron and placebo groups at 3 months in patients with dry AMD (P = 0.03) compared to wet AMD (P = 0.007), but these inter-group differences were no longer significant at 6 months (P = 0.12 and P = 0.26, respectively).

**Figure 3:**
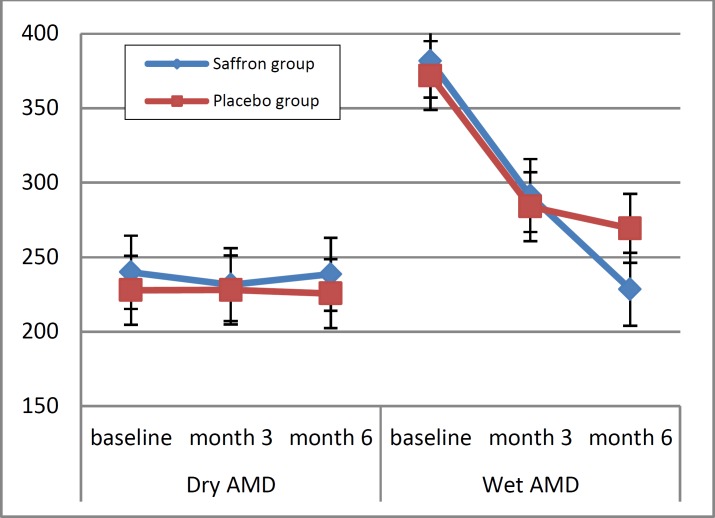
Trend of changes in electroretinogram amplitude (Mvolt) from baseline to 6 months in the treatment (saffron) and control (placebo) groups of patients with dry and wet age-related macular degeneration (AMD).

Figure 3 illustrates the trend for changes in ERG amplitude. According to the results of the repeated measures analysis, a significant effect between the saffron and placebo groups was only observed for ERG amplitude in patients with dry AMD (P = 0.05). No major side effects were observed among patients in either the saffron or the placebo group, and no patient reported severe complications, such as bleeding.

**Table 1:** Inter-group comparisons of macular thickness (micron) and electroretinogram (ERG) amplitude (Mvolt) from baseline to 6 months in the treatment (saffron) and control (placebo) groups of patients with dry and wet age-related macular degeneration (AMD).

	**Dry AMD**				**Wet AMD**			
	**Baseline**	**3 months**	**6 months**	P value*	**Baseline**	**3 months**	**6 months**	**P value** *
**Macular thickness **				0.58				0.12
**Saffron group**	227.92 ± 31.5	228 ± 28.3	225.64 ± 30.3		371.83 ± 41.3	283.87 ± 4.3	269.25 ± 3.7	
**Placebo group**	239.87 ± 37.4	231.6 ± 24.6	238.54 ± 22.3		381.55 ± 48.5	291.33 ± 4.9	228.47 ± 8.9	
**P value** **	0.32	0.58	0.28		0.49	0.84	0.05	
**ERG amplitude**				0.05				0.21
**Saffron group**	100.68±31.3	102.14 ± 3.1	102.9 ± 3.1		80.45 ± 3.5	80.97 ± 0.27	81.53 ± 2.8	
**Placebo group**	85.88 ± 35.4	89.8 ± 3.5	89.6 ± 3.5		76.38 ± 2.1	79.92 ± 0.21	79.42 ± 2.1	
**P value** **	0.20	0.03	0.12		0.63	0.007	0.26	

* P value for the effect of time in the repeated measures analysis of variance;

** P value for group comparisons with independent t-tests.

## DISCUSSION

In this study, we demonstrated the efficacy of saffron supplementation for improving retinal function after 3 months of daily treatment in patients with AMD. In addition, our results suggest that saffron supplementation, when combined with intravitreal bevacizumab, may significantly improve macular thickness in patients with wet AMD after 6 months of daily treatment. Our results are in agreement with other studies with similar follow-up durations that showed a significant improvement in retinal function in association with antioxidant supplementation (30-35). In addition, the results of the present study may be confidently attributed to saffron supplementation since the distribution of known risk factors (i.e., smoking) was similar in both groups. In our study, ERG amplitude recordings were significantly increased in the saffron group after 3 months of treatment, but these changes were not significant at 6 months. According to other studies, (36-39) response to treatment in patients with retinal degenerative disease is often initially impressive but usually declines with time, so the results of our study are consistent with this trend. We can assume that the improvements in retinal function observed in this study are related to the activity of chemical components of saffron, specifically to the important role of crocin, and antioxidant derivatives of carotenoids. We hypothesize that the ERG changes observed in the present study indicate that mid-term saffron supplementation may reduce photoreceptor damage induced by chronic oxidative injury. (39-41). Based on existing hypotheses, these components have the potential to act as protective factors against oxidative damage for aging and AMD retinas, whose disease pathophysiology has been linked to light-induced oxidative damage to the outer retina (27, 28, 40).

In patients with WET type AMD, macular thickness decreased after 3 months and after 6 months in both the saffron and placebo groups. This reduction could be explained by the use of intravitreous bevacizumab (IVB), but these changes were significant in the saffron group, especially in the sixth month. The reason for this increased reduction in macular thickness is not clear, however, but we hypothesize that the neuroprotective properties of saffron or the action of saffron on retinal pigment epithelial (RPE) cells may play a role. Therefore, more extensive research at the cellular level is warranted to reveal the mechanism behind this phenomenon. In our study, objective improvement in visual acuity was not found in either the saffron or the placebo group, although patients in the saffron group frequently gave subjective reports of better vision as well as reports of increased general wellbeing. As a result, patients in this treatment group stated that they planned to continue taking the drug for longer periods. This may be related to the reported antidepressant properties of saffron. Since it has been shown that AMD is associated with depression (30% of patients with AMD are depressed), (32, 42) saffron may serve a dual purpose in this patient population. Changes in the severity of AMD related to saffron supplementation at the end of the study period. We found that patients with a more advanced disease state received a greater benefit as evidenced by significant changes in ERG findings. However, these changes could not be proven statistically due to the small sample size. As such, future studies with a larger sample size are required. In conclusion, daily supplementation with 30 mg of saffron for 6 months was associated with statistically significant changes in macular OCT and ERG parameters in patients with both dry and wet AMD. Saffron supplementation may induce a mid-term, significant improvement in retinal function in AMD. The authors propose further research regarding saffron supplementation in clinical practice.
